# Revised Diffusion Law Permits Quantitative Nanoscale Characterization of Membrane Organization

**DOI:** 10.1021/acs.analchem.5c00021

**Published:** 2025-05-29

**Authors:** Barbora Svobodová, David Št’astný, Hans Blom, Ilya Mikhalyov, Natalia Gretskaya, Alena Balleková, Erdinc Sezgin, Martin Hof, Radek Šachl

**Affiliations:** † 86875 J. Heyrovský Institute of Physical Chemistry of the Czech Academy of Sciences, Dolejškova 3, Prague 182 23, Czech Republic; ‡ Faculty of Mathematics and Physics, Charles University, Ke Karlovu 5, Prague 121 16, Czech Republic; § Department of Physical and Macromolecular Chemistry, Faculty of Science, Charles University, Hlavova 8, Prague 128 40, Czech Republic; ∥ Science for Life Laboratory, Department of Applied Physics, Royal Institute of Technology, Solna 17165, Sweden; ⊥ Science for Life Laboratory, Department of Women’s and Children’s Health, 27106 Karolinska Institutet, Tomtebodavägen 23, Solna 17165, Sweden; # Shemyakin-Ovchinnikov Institute of Bioorganic Chemistry of the Rusian Academy of Science, Moscow 117997, Russia

## Abstract

The formation of functional nanoscopic domains is an inherent property of plasma membranes. Stimulated emission depletion combined with fluorescence correlation spectroscopy (STED-FCS) has been previously used to identify such domains; however, the information obtained by STED-FCS has been limited to the presence of such domains while crucial parameters have not been accessible, such as size (*R*_d_), the fraction of occupied membrane surface (*f*), in-membrane lipid diffusion inside (*D*_in_) and outside (*D*_out_) the nanodomains as well as their self-diffusion (*D*_d_). Here, we introduce a quantitative approach based on a revised interpretation of the diffusion law. By analyzing experimentally recorded STED-FCS diffusion law plots using a comprehensive library of simulated diffusion law plots, we extract these five parameters from STED-FCS data. That approach is verified on ganglioside nanodomains in giant unilamellar vesicles, validating the Saffman-Delbrück assumption for *D*_d_. STED-FCS data in both plasma membranes of living PtK2 cells and giant plasma membrane vesicles are examined, and a quantitative framework for molecular diffusion modes in biological membranes is presented.

## Graphical abstract

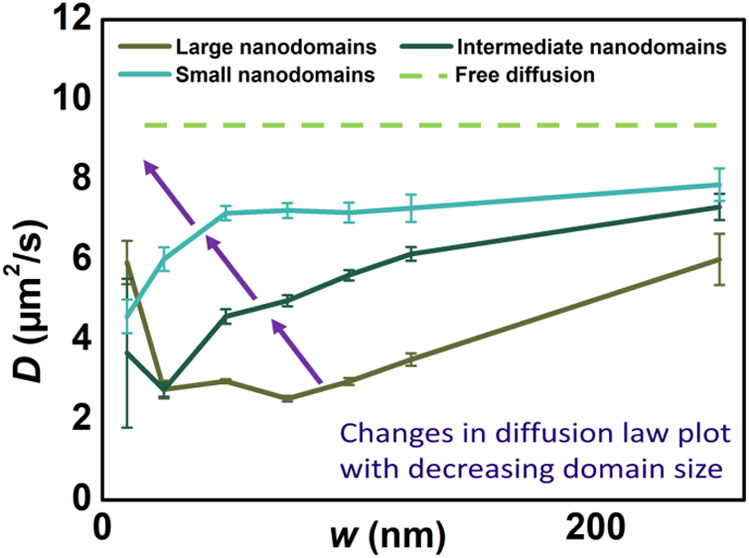

## Introduction

Thanks to its excellent spatiotemporal resolution, stimulated emission depletion combined with fluorescence correlation spectroscopy (STED-FCS) allows adequate diffusion law plot analysis^1^ at the nanoscale for which it has become a promising tool to study lipid dynamics within nanoscopically heterogeneous membranes.^2–14^ In this approach, the apparent diffusion coefficient (*D*) of a fluorescently labeled molecule diffusing in the membrane is typically plotted against the spot radius (*w*) (Figure 1A,B).^15,16^ For optimal sensitivity, the spot radius should approach or be smaller than the size of the obstacles being studied. In the case of nanosized membrane obstacles, this condition leads to what we call STED-FCS diffusion law plot dependencies, where the diffusion coefficient is measured for waist radii ranging between 20 and 250 nm. These diffusion law plots typically show a specific pattern, depending on the type of interactions and obstacles in the lipid bilayer (Figure 1A). The complexity of lipid dynamics generates exceptions to this rule, though, and occasionally one can get the same pattern for several diffusion modes.^17^

**Figure 1:**
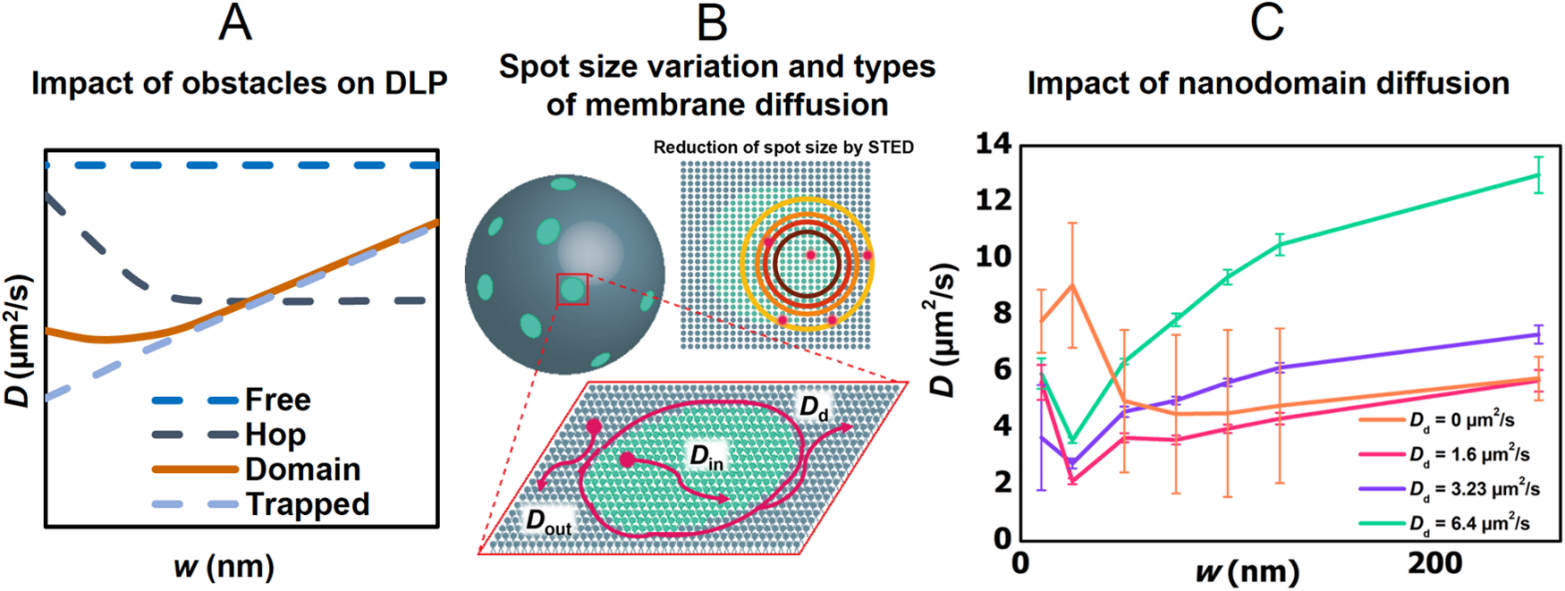
(A) Computationally generated diffusion law plots (DLP) depicting the relationship between the probe diffusion coefficient (*D*) and the radius of the illuminated focal spot (*w*). The shape of these plots reflects the type of obstacles in the membranes: Free diffusion in homogeneous membrane areas shows a constant *D*, independent of the spot radius (dashed dark blue line); trapped diffusion occurs when molecules are temporarily immobilized, resulting in a decreasing *D* as *w* decreases (dashed light blue line); hop diffusion involves movement within a meshwork structure, with fast diffusion over short distances and impeded diffusion over longer distances (dashed dark gray line). The diffusion in the presence of immobile nanodomains leads to a gradual decrease of *D* with decreasing *w* and flattening of this dependence for small waist radii (solid orange line). (B) In STED-FCS, the effective spot size is reduced by applying stronger STED laser pulses. For membranes with mobile nanodomains, three diffusion types can exist: probe diffusion inside (*D*_in_) and outside (*D*_out_) nanodomains, and diffusion of nanodomains themselves (*D*_d_). (C) Diffusion law plots for nanodomains with increasing mobility: static nanodomains (*D*_d_ = 0) and mobile nanodomains (*D*_d_ = 1.6 – 6.4 μm^2^/s).

Interpretations of diffusion data have so far relied on assumptions that are not always universally accepted. Particularly, in cellular membranes with nanoscopic domains, these domains were assumed to be static despite their generally dynamic nature, which may affect the interpretation of the results (Figure 1C).^1,17–20^ In this work, we incorporate nanodomain mobility into simulations of probe diffusion in nanoscopically heterogeneous membranes and generate a comprehensive library of STED-FCS diffusion law plots under various condition. Analysis of these in-silico plots reveals distinct characteristic patterns that serve as fingerprints, indicating the size (*R*_d_) and surface concentration (*f*) of the nanodomains. To validate this fingerprint analysis, we apply it to STED-FCS data collected from a well-defined model system: membranes containing ganglioside GM_1_ nanodomains of known *R*_d_ and *f*. Additionally, we leverage the generated STED-FCS diffusion law library to quantitatively analyze these experimental plots, enabling access to lipid dynamics both within and outside the nanodomains. Finally, using the STED-FCS library, we revisit previously published diffusion law plots for fluorescently labeled ganglioside GM_1_ in both cellular plasma membranes and giant plasma membrane vesicles.^19^ This reassessment not only confirms the validity of our experimental approach but also uncovers previously inaccessible information in STED-FCS diffusion law plots, significantly enhancing the interpretative framework for STED-FCS data in the context of mobile membrane nanodomains.

## Experimental Section

### In-Silico Generation of STED-FCS Diffusion Law Plots for Mobile Nanodomains

To generate STED-FCS diffusion law plots for lipid membranes with mobile nanodomains, we employed Monte Carlo (MC) simulations as described in detail in^1,20^ and summarized in Supporting Information (SI).

### Quantitative Analysis of STED-FCS Diffusion Law Plots

For the quantitative analysis of the STED-FCS diffusion law plot dependencies, we generated a comprehensive set of diffusion law plots for different combinations of simulation input parameters, including the diffusion coefficients for nanodomain self-diffusion, *D*_d_, and for probe diffusion in nanodomains, *D*_in_, the probe distribution coefficient between the nanodomains and the remaning bilayer, *K*_d_, the nanodomain radius, *R*_d_, and the fraction of the membranbe surface occupied by nanodomains, *f*. The complete library of these generated dependencies is available at the following link: [https://doi.org/10.48700/datst.sg1fq-8rc76] and can be used to fit experimentally obtained STED-FCS diffusion law plots. See also Supporting Information for more information about the library.

Our fitting approach involved comparing experimentally measured diffusion law plots with the generated diffusion dependencies by calculating a chi-squared parameter to characterize their similarity. We analyzed the chi-squared values only for the physically realistic combinations of all parameters. For the diffusion dependencies of giant unilamellar vesicles (GUVs), the parameters *R*_d_ and *f* were determined independently using MC-FRET, while *D*_out_ was experimentally measured by FCS (9 ± 1 μm^2^/s and 9.5 ± 1 μm^2^/s for free diffusion in DOPC/Chol (75/25) and in DOPC/SM (90/10), respectively^21^). Our goal was to identify in the library such a diffusion law plot that best matched the experimental data by testing various combinations of *D*_d_, *D*_in_, and *K*_d_. Since the initial match was unsatisfactory, we revisited the library to optimize *D*_d_, *D*_in_, *K*_d_, and *f*, allowing only adjustments to *f* in the range *f* = 0.3–0.5.

In the case of giant plasma membrane vesicles (GPMVs) and cell plasma membranes, the situation differed because neither the size of the nanodomains nor the fraction of surface coverage by nanodomains was known in advance. Therefore, in this case, we primarily focused on the determination of these two characteristic parameters. Based on the GUV experiments presented below, we made the following assumptions: (1) The nanodomains move according to the Safmann-Delbrück model, where *D*_d_ depends on the size of the nanodomains and the mobility of the molecules in the surrounding environment (*D*_out_). (2) GM_1_-Atto565 has a relatively high affinity for existing nanodomains, characterized by *K*_d_ ∼ 5. Finally, (3) the mobility of GM_1_-Atto565 within nanodomains is half that of the mobility in the surrounding nondomain phase. These assumptions allowed us to reduce the number of optimized parameters to *R*_d_ and *f*, leaving *D*_d_, *K*_d_, and *D*_in_ fixed. We also attempted to optimize *D*_in_ in the final step, but this optimization did not improve the fit in the investigated range of physically acceptable *D*_in_ values (from *D*_in_ = *D*_out_/3 to *D*_in_ = *D*_out_/1.5), confirming *D*_in_ = *D*_out_/2.

### STED-FCS Experiments on GUVs: Methodology

STED-FCS measurements (5 s each) were performed at the bottom of GUVs with a Leica SP8 FALCON FLIM/FCS microscope. The GM1-Atto565 probes were excited at 561 nm selected from the tunable pulsed white light laser, and fluorescence was depleted using a high-power continuous-wave STED red laser at 660 nm. Built-in excitation and depletion laser filters were used to block laser scattering, and optimized dichroic mirrors filtered out emission. The Atto565 emission was recorded at 575–635 nm by tuning the mechanical slit in front of the Leica HyD-SMD detector. No hardware gating was set as all photon time-filtering was done in the FALCON mode.^22^ For the calibration of the spot size, measurements of DOPC GUVs with different STED laser powers were utilized. Assuming free diffusion allows the spot size calculation according to the following equation:

1 $$w_{\mathrm{STED}}=w_{\mathrm{confocal}}\sqrt{\tau_{\mathrm{STED}}/\tau_{\mathrm{confocal}}},\;w_{\mathrm{confocal}}=\sqrt{D4\;\tau_{\mathrm{confocal}}}$$

where *w* is the radius of the spot size and τ_confocal_, τ_STED_ the transit times which the molecule spends in the observation spot in confocal and at a certain STED laser power, respectively. Individual ACFs were fitted with eq S2 in the Supporting Information.

## Results and Discussion

To exploit the full potential of STED-FCS diffusion law plots and ensure their unbiased interpretation, our primary goal was to pinpoint distinctive trends within these plots that could signify key nanodomain features, such as *R*_d_ and *f*. We thus initiated our study by generating a series of these dependencies in-silico. This involved exploring various combinations of parameter values that describe the diffusion of fluorescent lipid probes in the presence of mobile nanodomains. Specifically, we kept the diffusion coefficient of lipids outside the nanodomains (*D*_out_) using previously determined values for labeled GM_1_ in GUVs.^21^ The sizes of the nanodomains (*R*_d_) were chosen to match the sizes commonly encountered in biological membranes: (1) small nanodomains with a radius of 25 nm; (2) intermediate-sized nanodomains with a radius of 75 nm; and (3) large nanodomains with a radius of 120 nm. The surface concentration of nanodomains (*f*) was chosen to range from 10 to 50%, with the upper limit approximately determined by the maximum number of nanodomains that can still be placed side by side so as not to overlap. Regarding 2-dimensional nanodomain movement, our simulations exclusively considered nanodomains moving with a diffusion coefficient (*D*_d_) calculated using the Saffman-Delbrück model,^23^ a choice supported by our later experimental findings in this work. Additionally, we anticipated that the shape of the diffusion law plots could be significantly influenced by the partition coefficient of probes in the nanodomains (*K*_d_), as well as the probe diffusion rate within the nanodomains (*D*_in_). Consequently, we expanded the set of generated diffusion law plots to include these dependencies as well. However, in view of our previous results,^20^ which demonstrated that the shape of diffusion plots is significantly less affected by the presence of nanodomains when *K*_d_ ≤ 1, we focus this manuscript exclusively on the case where *K*_d_ ≥ 1. The results obtained are summarized in Figure 2, with a comprehensive set of generated diffusion law plot dependencies available in Figure S1 in the Supporting Information file.

**Figure 2:**
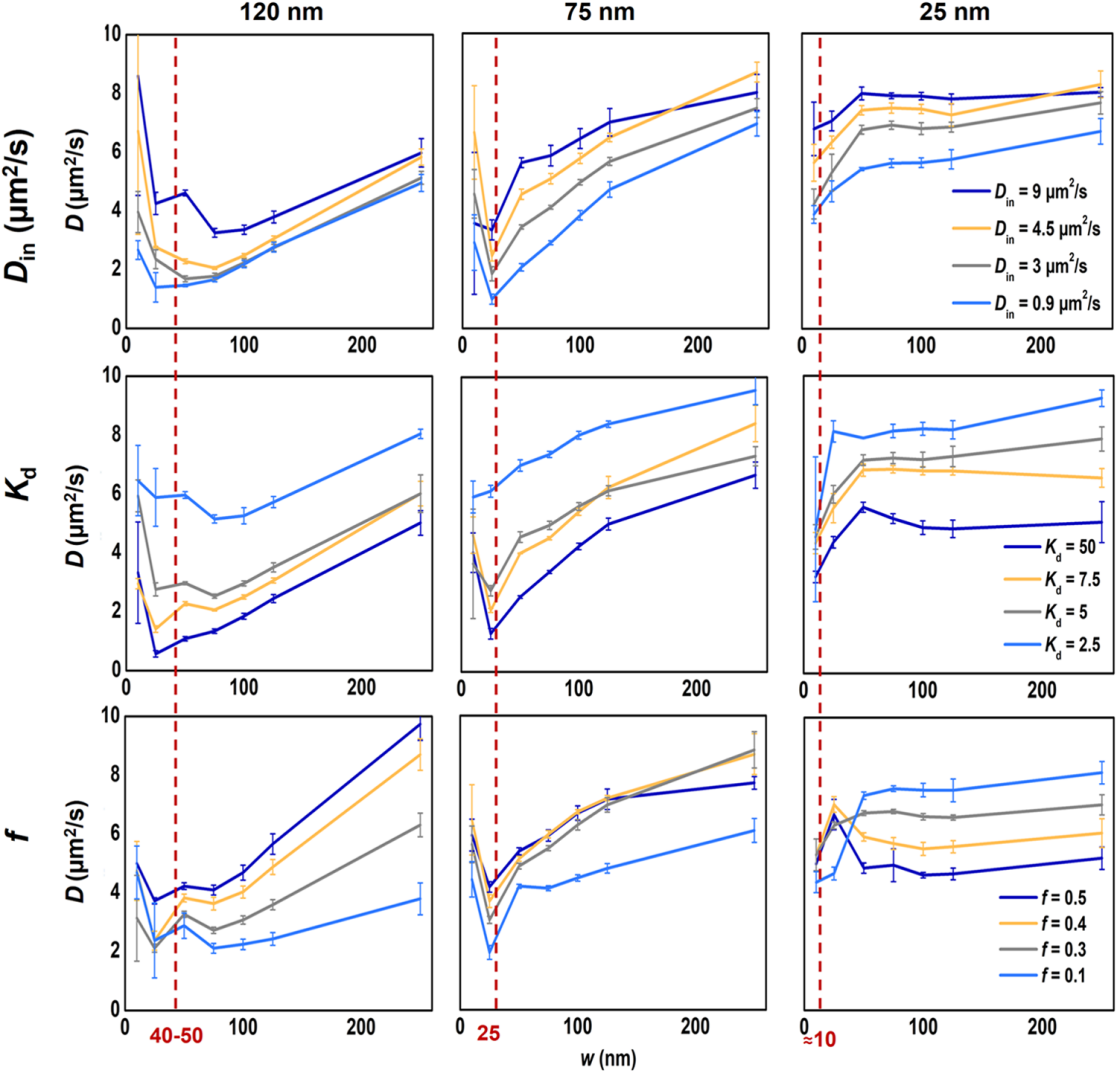
Computationally generated STED-FCS diffusion law plots illustrating mobile nanodomains with *D*_d_ modeled according to the Saffman-Delbrück model. Plots are shown for three nanodomain sizes: large (left column), intermediate (middle column), and small (right column). The impact of the probe diffusion coefficient within the nanodomains (*D*_in_) is depicted in the upper row, the probe distribution coefficient between the nanodomains and the surroundings (*K*_d_) is depicted in the middle row, and the area fraction (*f*) occupied by the nanodomains is depicted in the lower row. If not stated otherwise, *D*_in_ = 4.5 μm^2^/s, *K*_d_ = 5, and *f* = 0.25 *D*_out_ = 9 μm^2^/s. The red line indicates the minimum in the diffusion law plot corresponding to approximately one-third of the *R*_d_. Extended data set is shown in Figure S1.

Analyzing trends in these dependencies revealed several indicators of nanodomain properties (Figure 3). The diffusion law plots exhibit a characteristic shape reminiscent of an asymmetric funnel profile, with a broader shoulder extending toward larger focal waist radii. The minimum point of this funnel appears to correspond to approximately one-third of the nanodomain radius *R*_d_ (highlighted by red-dashed lines in Figure 2), while its depth is determined by *K*_d_ and *D*_in_. Stronger entrapment and retardation of probe movement within nanodomains lead to deeper minima in the plots. Meanwhile, *R*_d_ dictates the extent of this characteristic shape captured in our in-silico analysis. The large nanodomains (*R*_d_ = 120 nm) exhibit a strong funnel profile dependence that is missing the final plateau in the waist radius range of 20–250 nm, whereas the small ones (*R*_d_ = 25 nm) reveal only the final plateau of the extended funnel arm. This analysis thus highlights characteristic fingerprinting of the nanodomain size in the diffusion law plots, mirrored in the position of the minimum in the funnel-shaped dependence and the segment of this dependence captured (Figures 3A and S1).

**Figure 3:**
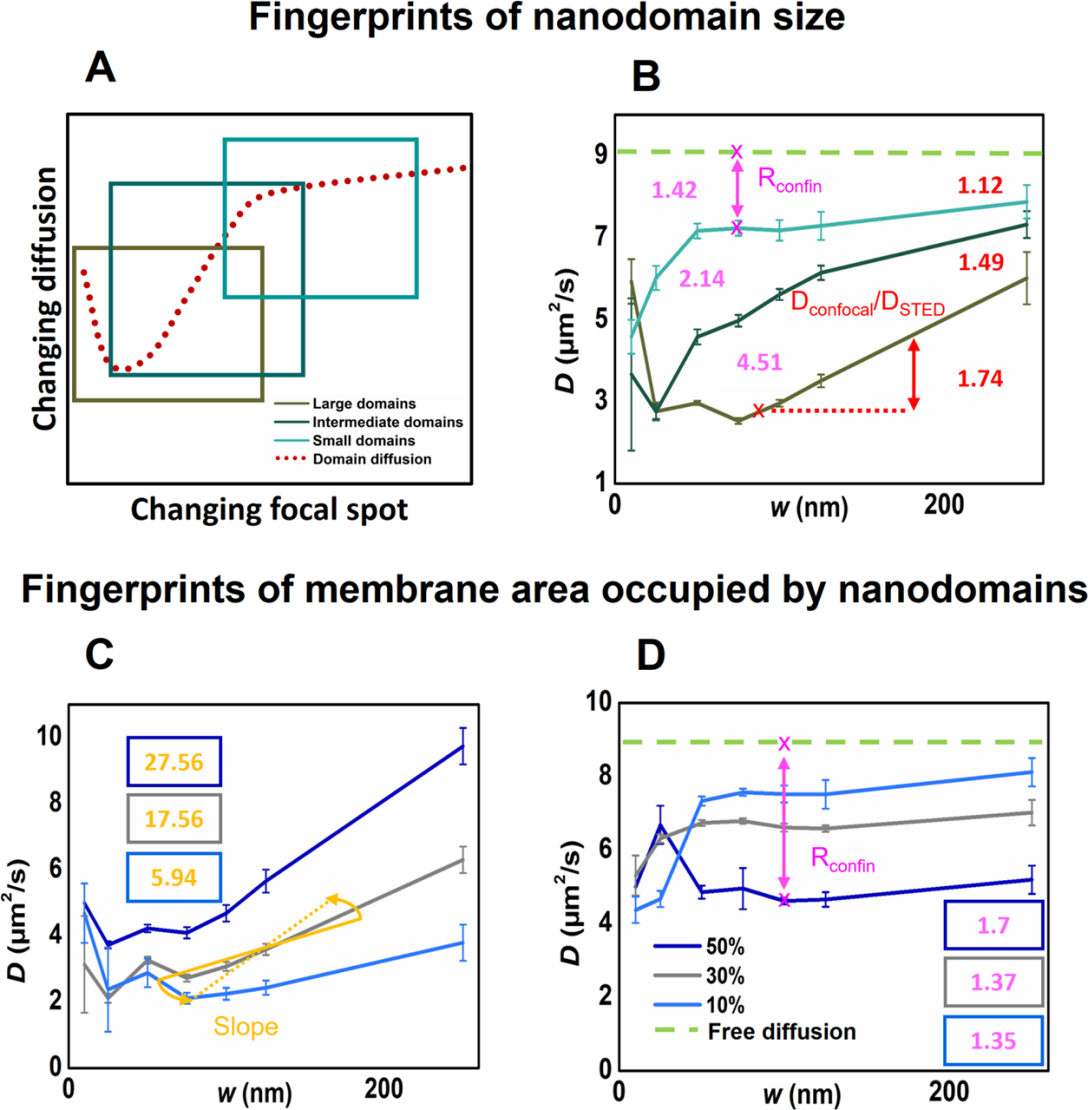
(A and B) Identified fingerprints based on performed simulations indicative of nanodomain size. (A) Diffusion law plot in the presence of moving nanodomains exhibits a characteristic shape resembling an asymmetric funnel. The nanodomain size determines the extent of this characteristic shape observed in the experiment. Large nanodomains (*R*_d_ = 120 nm) display a funnel-like dependence lacking a final plateau in the waist radius range of 20–250 nm (dark brown); intermediate-sized nanodomains (*R*_d_ = 75 nm) show a rising portion of the funnel that gradually levels off (dark green), while small nanodomains (*R*_d_ = 25 nm) reveal only the final plateau of the extended funnel shape (cyan). (B) Ratiometric indicators of nanodomain size: *D*_confocal_/*D*_STED_ ratio (depicted in red) derived from the probe diffusion coefficients *D* determined for *w* = 160 nm and *w* = 60 nm. *D*_confocal_/*D*_STED_ > 1.8 indicates the presence of large domains, *D*_confocal_/*D*_STED_ ∈ (1.2; 1.6) suggests intermediate-sized nanodomains, while *D*_confocal_/*D*_STED_ < 1.2 suggests small nanodomains or a homogeneous membrane. Confinement ratio *R*_confin_ = *D*_(no entrap.)_/*D*_60_ (depicted in magenta) expressing the reduction in diffusion rate attributed to nanodomain formation, where *D*(no entrap.) is the diffusion coefficient of a probe showing no entrapment in nanodomains, and *D*_60_ is the diffusion coefficient of a probe confined within nanodomains and measured with the beam spot radius *w* = 60 nm. *R*_confin_ > 2.8 indicates the presence of large domains, while *R*_confin_ ∈ (1.7; 2.8) suggests intermediate-sized nanodomains. *R*_confin_ < 1.7 suggests small nanodomains or a homogeneous membrane. (C and D) Identified fingerprints based on performed simulations indicative of nanodomain fraction *f* shown for both large (C) and small nanodomains (D). *K*_d_ = 5 and *D*_in_ = 4.5. (C) In the case of large nanodomains, the slope of the diffusion law plot dependence calculated as *S* = (*D*_160_–*D*_60_)/(*w*_160_–*w*_60_) emerges as the primary indicator of *f*. This slope exhibits a low value when *f* = 0.1 (*S* ≈ 5), increasing to as high as *S* ≈ 27 for *f* = 0.5. (D) In the case of small nanodomains, *f* is primarily indicated by *R*_confin_ increasing from *R*_confin_ ≈ 1.3 for *f* = 0.1 to *R*_confin_ ≈ 1.7 for *f* = 0.5. As anticipated, a higher *f* results in a more pronounced confinement. For a homogeneous membrane, *D*_160_/*D*_60_ = *R*_confin_ = *S* = 1. With mobile nanodomains, these values are greater than 1.

In order to simplify the analysis of STED-FCS data, Sezgin et al. introduced a simplified approach centered on the analysis of a single ratiometric parameter *D*_confocal_/*D*_STED_.^19^ This diffusion coefficient ratio is derived from the probe diffusion coefficients *D* determined for two focal spot sizes: *w* = 160 nm, denoted as *D*_confocal_ (representing the confocal spot size in our case), and *w* = 60 nm, denoted as *D*_STED_ (a waist radius size typically achievable with STED microscopy) (see also Figure 3B). Widely adopted in literature, the parameter *D*_confocal_/*D*_STED_ acts as a tentative indicator of nanoscale membrane heterogeneity, essentially reflecting the rate of diffusion retardation relative to the focal spot size *w*.^3,13,16,19,24^ This ratio is consolidated in a part of Table S2 and Figure 3B, supplementing the diffusion dependencies illustrated in Figure 2. In a homogeneous membrane devoid of obstacles, *D*_confocal_/*D*_STED_ = 1. Notably, Table S2 demonstrates that *D*_confocal_/*D*_STED_ also holds a strong predictive value regarding the nanodomain size. Specifically, a *D*_confocal_/*D*_STED_ value exceeding 1.8 robustly indicates the presence of large domains, except in rare instances of exceptionally high *K*_d_ or extremely slow diffusion within the nanodomains. Conversely, an *D*_confocal_/*D*_STED_ value falling within the range 1.2–1.6 suggests intermediate-sized nanodomains, while an *D*_confocal_/*D*_STED_ value below 1.2 suggests small nanodomains or a homogeneous membrane without obvious barriers.

To differentiate between the latter cases, one can assess the reduction in diffusion rate attributed to nanodomain formation using an additional parameter defined as *R*_confin_ = *D*_no entrap_/*D*_60_ and called the confinement ratio (Figure 3B). Even with minimal probe entrapment in nanodomains, this ratio notably deviates from 1, even in the presence of small domains (Table S2). Although it appears to offer superior sensitivity to nanodomain size as compared to *D*_confocal_/*D*_STED_, obtaining it necessitates an additional measurement of *D*_no entrap_ in a membrane environment where no probe entrapment occurs. This can be done either in membranes devoid of obstacles or using fluorescent probes that do not become trapped in nanodomains. Overall, our in-silico analysis shows that STED-FCS diffusion law plots generated for mobile nanodomains contain a variety of signatures that are indicative of the presence and approximate size of the detected nanodomains (Figure 3).

In fact, our in-silico analysis also predicts the shape of the diffusion law plot dependencies to be largely influenced by the surface concentration of nanodomains *f*. However, interpreting these dependencies becomes more complex because the response of the diffusion law plot to *f* relies heavily on *R*_d_, while the influence of either *K*_d_ or *D*_in_ is relatively minor (Figure 2). Fundamentally, two distinct scenarios emerge in diffusion law plots, each offering unique fingerprints of the nanodomain fraction, contingent upon nanodomain size: In the case of large nanodomains, *f* is distinctly determined by the slope (*S*) of the respective dependence (*S* = (*D*_160_–*D*_60_)/(*w*_160_–*w*_60_)) (Figure 3). Conversely, for small nanodomains, the confinement ratio emerges as the primary indicator of *f* (Figure 3D).

To experimentally validate and utilize the nanodomain indicators identified through computational simulations, we turned in the next step to an inherently nanoscopically heterogeneous system of GUVs containing physiologically relevant amounts of ganglioside GM_1_.^25–32^ We recorded STED-FCS diffusion law plots using GM_1_ labeled in the headgroup with Atto565 as a lipid tracer (referred to as GM_1_-Atto565). Gangliosides exhibit a pronounced tendency to segregate into lipid nanodomains spanning 10–120 nm in radius, influenced by the specific ganglioside type and the composition of the surrounding environment.^25–28,33,34^ Recently, we also characterized this system in detail using the Monte Carlo Förster Resonance Energy Transfer (MC-FRET) technique, which determines both the radius and surface concentration of these formed nanodomains.^35–38^ Consequently, we possessed a well-defined model system with ganglioside nanodomains of known *R*_d_ and *f*.^26,27^ Our primary objective extended beyond merely applying identified qualitative indicators to an experimental system featuring mobile nanodomains. We sought to conduct a thorough quantitative analysis of the STED-FCS diffusion law plots, a task not previously undertaken for such systems.

For our experiments, we chose GUVs containing two different types of nanodomains: (a) large nanodomains with *R*_d_ = 120 ± 20 nm and nanodomain surface coverage *f* = 0.5 ± 0.1 ^26,27^ formed in 1,2-dioleoyl-*sn*-glycero-3-phosphocholine (DOPC)/cholesterol (Chol)/GM_1_ bovine brain sodium salt (GM_1_) (75/25/4 mol %) mixtures, where the nanodomain size matched perfectly the size of large nanodomains characterized in-silico; and (b) small nanodomains formed in DOPC/N-stearoyl-D-erythro-sphingosylphosphorylcholine (SM)/GM_1_ (90/10/4 mol %) mixtures that according to the previously performed MC-FRET analysis had a radius of 23 ± 20 nm and occupied 47 ± 17% ^26,27^ of the bilayer surface. As a result, these nanodomains had a size comparable to the smallest waist diameter we could accomplish in our experimental setup (*d*_STED_^min^ = 60 nm) and resembled in size small nanodomains used in the simulations.

The experimental results in Figure 4 display STED-FCS diffusion law plots for both large and small GM_1_ nanodomains utilizing GM_1_-Atto565. With this fluorescent label, we achieved STED focal spot sizes ranging from 60 to 160 nm. This spatial range allowed for a comprehensive quantitative analysis, although it was not large enough to fully capture the funnel-shaped dependency, even for large nanodomains (compare Figure 3A with Figure 4).

**Figure 4:**
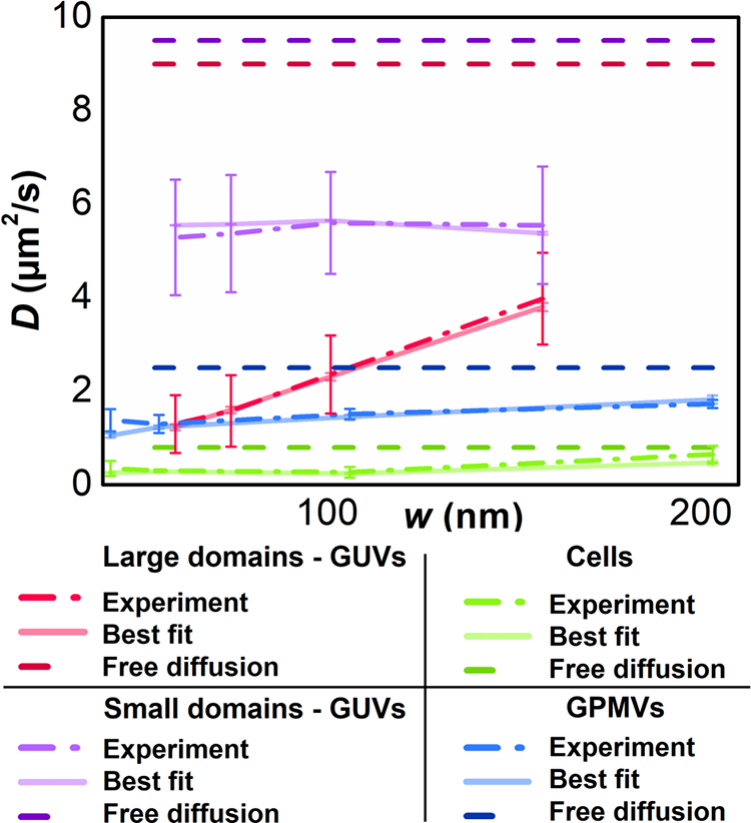
Experimentally obtained STED-FCS diffusion dependencies (dashed-dotted lines) and their best fits (solid lines) for GM_1_-Atto565 diffusion in: DOPC/Chol/GM_1_ (75/25/4) GUVs containing large nanodomains (*R*_d_ = 120 nm, red) and DOPC/SM/GM_1_ (90/10/4) GUVs containing small nanodomains (*R*_d_ = 23 nm, violet). The figure also shows experimentally measured diffusion dependencies (adopted from^19^) and their best fits for GM_1_-Atto647N diffusing in: the plasma membranes of PtK2 cells (green) and in GPMVs made from these cells (blue). In this case, quantitative analysis of STED-FCS diffusion law plots was used to determine *R*_d_, *f*, and *D*_in_. For comparison, limiting cases of free diffusion with a waist-independent diffusion coefficient value corresponding to *D*_out_ are shown in the Figure (dashed lines).

Importantly, the diffusion law plots for both large and small nanodomains exhibited characteristic features consistent with our simulations of probe diffusion in the presence of mobile nanodomains. Specifically, the diffusion law plot pattern for large nanodomains showed a sharp increase without fully reaching a plateau (compare Figure 3A with Figure 4). Moreover, the introduction of simplified ratiometric parameters *D*_160_/*D*_60_ = 3.07 ± 2.21 and *R*_confin_ = 6.94 ± 4.01 confirmed the presence of nanodomains with an estimated radius of approximately 120 nm (Figure 3B and Table S2). Evaluating the diffusion law slope parameter, *S* = 27.35 ± 5.38, suggested a surface concentration of nanodomains with a fraction *f* greater than ∼ 0.3 (Figure 3C and Table S2), aligning with our assumptions based on MC-FRET experiments.^26,27^ In contrast, the diffusion law plot pattern for small nanodomains appeared flat with the diffusion ratio *D*_160_/*D*_60_ = 1.04 ± 0.48, indicating the presence of either very small nanodomains or a homogeneous membrane (Figure 3B with Table S2). A homogeneous membrane was unlikely given that the confinement ratio *R*_confin_ = 1.79 ± 0.61 deviated significantly from 1. This high *R*_confin_ value further suggests a high surface concentration of very small nanodomains (*f* ∼ 0.4), consistent with our previous MC-FRET results (compare Figure 3D with Table S2).^26^ In summary, the observed patterns in our experimentally recorded STED-FCS diffusion law plots, as depicted in Figure 4, closely match the fingerprints predicted by our MC simulations. This alignment validates the robust application of STED-FCS diffusion law plots to nanoscopically heterogeneous membrane systems featuring dynamic nanodomains, paving the way for quantitative analyses of diffusion law plot dependencies.

To quantitatively analyze the STED-FCS diffusion law plots in the presence of GM_1_ nanodomains (Figure 4), we generated an extended set of diffusion law plot dependencies using various combinations of simulation input parameters. This library (accessible via this link: [https://doi.org/10.48700/datst.sg1fq-8rc76]) serves as a straightforward analytical tool for identifying the parameter combination that best matches the experimental data, determined by the lowest value of the reduced chi-squared parameter (link: [https://doi.org/10.48700/datst.sg1fq-8rc76]).

Our analysis specifically targeted the STED-FCS diffusion law plots for GM_1_ nanodomains within DOPC/Chol/GM_1_ (75/25/4 mol %) or DOPC/SM/GM_1_ (90/10/4 mol %) GUVs, particularly examining the diffusion law plots for large and small nanodomains as depicted in Figure 4. This iterative process enabled us to determine optimal values of *D*_d_, *D*_in_, and *K*_d_ for both large and small GM_1_ nanodomains. The values obtained, with *D*_d_ = 2.6 ± 1.3 μm^2^/s for large nanodomains and *D*_d_ = 4.8 ± 2.4 μm^2^/s for small nanodomains, confirm the high mobility of GM_1_ nanodomains within GUV membranes, consistent with the Saffman-Delbrück model (implying that *D*_d_ is a function of *R*_d_). Furthermore, a *K*_d_ value of 10 ± 5 for both large and small nanodomains indicates that individual ganglioside molecules are predominantly localized within nanodomains with only minimal occurrence outside these domains in the membrane. This finding underscores GM_1_’s strong tendency to segregate spatially into nanodomains.^25–27,31,33,39–41^ Our approach facilitates the examination of lipid diffusion rates within nanodomains, a parameter that is typically challenging to determine directly. Our analysis revealed that *D*_in_(GM_1_ – Atto565) = 4.5 ± 1.5 μm^2^/s in both small and large nanodomains, which is only twice as slow as the diffusion coefficient recorded for homogeneous DOPC/Chol/GM_1_ (75/25/4) membranes. This indicates that the retarding effect of the packed ganglioside environment is only mildly supporting the fluid and disordered character of GM_1_ nanodomains.^10^

In the final comparison analysis, we applied the developed quantitative procedure to re-examine previously published STED-FCS diffusion law plots for Atto647N-labeled ganglioside GM_1_ (GM_1_-Atto647N) in both plasma membranes of living cells and in giant plasma membrane vesicles (GPMVs) lacking cytoskeleton.^19^ These dependencies (Figure 4) were previously used primarily as evidence for gangliosides being localized into lipid nanodomains both in cellular plasma membranes and in GPMVs, with qualitative characterization of molecule diffusion based on diffusion law plots generated for static nanodomains. Given that the diffusion of GM_1_-Atto647N, unlike sphingomyelin or other lipids, is largely insensitive to the naturally occurring cytoskeleton in cellular plasma membranes causing detected hop diffusion;^19,42,43^ this system thus appears ideal for applying our developed quantitative analysis.

Following our previously outlined STED-FCS diffusion law plot analysis procedure, we first determined the ratiometric parameters *D*_confocal_/*D*_STED_ and *R*_confin_ for both cell membranes (*D*_confocal_/*D*_STED_ = 1.38 ± 0.57 and *R*_confin_ = 2.85 ± 1.21) and GPMVs (*D*_160_/*D*_60_ = 1.36 ± 0.27 and *R*_confin_ = 2.03 ± 0.90). The analysis of these parameters with help of Figure 3 and Table S2 allowed us to estimate the approximate size of the nanodomains in which GM_1_-Atto647N is entrapped. Specifically, assuming an increased affinity of GM_1_-Atto565 to the nanodomains (*K*_d_ ∼ 5–10), GM_1_ appears to be confined into intermediate to large nanodomains with *R*_d_ ≈ 75–120 nm in plasma membranes; whereas in GPMVs, it aggregates into intermediate-sized nanodomains with *R*_d_ ≈ 75 nm. To refine these estimates, we performed a quantitative analysis by identifying an in-silico generated diffusion law plot from the online library that best fits the experimentally obtained dependence (Figure 4, for more details, see **Materials and Methods**). This analysis revealed that in plasma cell membranes, GM_1_-Atto647N is segregated into nanodomains with a radius *R*_d_ = 120 ± 45 nm, *f* = 0.3 ± 0.2, and *D*_in_ = *D*_out_/2 = 0.4 ± 0.14 μm^2^/s. These GM_1_ nanodomain characteristics resemble those for GPMVs with *R*_d_ = 75 ± 45 nm, *f* = 0.2 ± 0.1, and *D*_in_ = *D*_out_/2 = 1.25 ± 0.4 μm^2^/s. Importantly, these nanodomain features are also consistent with independent MC-FRET experiments performed on DOPC/Chol/SM/GM_1_ (65/25/10/4) GUVs, which revealed GM_1_ nanodomains with *R*_d_ = 99 ± 20 nm and *f* = 0.5 ± 0.1.^26,27^ Furthermore, the results appear consistent also in terms of the recovered *D*_in_ coefficients that are consistently half the value of *D*_out_, indicating that the fluidity of the nanodomain interior is similar to that of the surrounding membrane phase.

## Conclusions

In summary, revising the diffusion law led to the development of a comprehensive library of simulated STED-FCS diffusion law plots under various conditions (see Figures 2 and S1 and the following link: [https://doi.org/10.48700/datst.sg1fq-8rc76]). In this study, we demonstrate how this library can be used to retrieve several key parameters from experimentally recorded STED-FCS diffusion law plots, providing insights into probe diffusion in the presence of mobile nanodomains. The retrieved parameters include nanodomain radius (*R*_d_), the fraction of occupied membrane surface (*f*), in-membrane lipid diffusion inside (*D*_in_) and outside (*D*_out_) the nanodomains, and their self-diffusion (*D*_d_).

The analysis of the simulated plots showed a prevailing asymmetric funnel-like profile influenced by *R*_d_, *D*_d_, and *f*. The mobility of lipids within the nanodomains (*D*_in_) and their affinity for the nanodomains (*K*_d_) only accentuated this characteristic shape. Based on these results, we identified *R*_d_ and *f* as the two key parameters shaping the final dependence, allowing for the identification of simple parameters to approximately estimate these parameters (Figure 3). Importantly, the previously published STED-FCS diffusion law plots,^3,19,24^ characterized by a gradual decrease in the diffusion coefficient *D* with decreasing focal spot waist radius *w* and flattening for small waist radii (Figure 1C), represent an incomplete dependence applicable only to large nanodomains with *R*_d_ ≥ 120 nm. The neglected increase in *D* with decreasing *w* for the smallest STED spot waists or the final plateau for the largest waists (Figure 2) was previously unshown.

Application of our analytical tool on GUV’s membranes with GM_1_ nanodomains, previously characterized experimentally by MC-FRET, confirms the Saffman-Delbrück model for nanodomain diffusion *D*_d_ and show that *D*_in_ to be only twice as slow as *D*_out_. These findings also hold for the diffusion of GM_1_ in membranes of GPMVs and PtK2 cells. The fact that, similar to the results for model membrane systems, fluid domains of about 120 nm size occupy a large fraction of the cell membrane (*f* = 0.3) indicates that extensive nanostructuring represents a generic phenomenon in ganglioside containing membranes. Overall, we believe that this new quantitative framework for diffusion law analysis will allow resolving the nanoscale architecture of other macromolecules in cells, enhancing the level of detail and information obtainable from STED-FCS data.

## Supplementary Material



## Notes

Materials and Methods including preparation of GUVs, tagging Atto565 to GM1, Monte Carlo simulations of probe diffusion and detailed information about the library of simulated STED-FCS diffusion law plots; additional results including ratiometric parameters calculated for various combinations of *R*_d_, *f*, *K*_d_, and *D*_in_, in-silico generated STED-FCS diffusion law plots and complementary α plots (PDF)
