# Supplementary material for: Revised Diffusion Law Permits Quantitative Nanoscale Characterization of Membrane Organization
Source: Anal Chem. 2025 May 29;97(22):11478–85. doi: 10.1021/acs.analchem.5c00021 (PMC12163893; doi:10.1021/acs.analchem.5c00021)
Supplement: Supplementary file 1 [file ac5c00021_si_001.pdf]

Supporting Information

Revised diffusion law permits quantitative nanoscale characterization of membrane organization

Barbora Svobodová[a,b], David Šťastný[a,c], Hans Blom[d,e], Ilya Mikhalyov[f], Natalia Gretskaya[f], Alena Balleková[a], Erdinc Sezgin[d,e,*] , Martin Hof[a,*] and Radek Šachl[a,*]

[a] J. Heyrovský Institute of Physical Chemistry of the Czech Academy of Sciences, Dolejškova 3, 182 23 Prague, Czech Republic; [b] Faculty of Mathematics and Physics, Charles University, Ke Karlovu 5, 121 16 Prague, Czech Republic; [c] Department of Physical and Macromolecular Chemistry, Faculty of Science, Charles University, Hlavova 8, 128 40 Prague, Czech Republic; [d] Science for Life Laboratory, Department of Applied Physics, Royal Institute of Technology, 17165, Solna, Sweden; [e] Science for Life Laboratory, Department of Women's and Children's Health, Karolinska Institutet, Tomtebodavägen 23, 17165 Solna, Sweden; [f] Shemyakin-Ovchinnikov Institute of Bioorganic Chemistry of the Russian Academy of Science, 117997, Moscow, Russia

[*]**Correspondence:**

Radek Šachl

radek.sachl@jh-inst.cas.cz

Martin Hof

martin.hof@jh-inst.cas.cz

Erdinc Sezgin

erdinc.sezgin@ki.se

Table of Content

Materials and Methods S2
- Materials S2
- Preparation of GUVs S2
- Tagging Atto565 to GM_1 .... S2
- Simulating two-dimensional probe movement in the membranes containing mobile nanodomains S3
- The library of in-silico generated STED-FCS diffusion law plots S5

Supporting results S6
- Trends in the anomalous coefficient alpha S8

Materials and Methods

Materials

1,2-dioleoyl-sn-glycero-3-phosphocholine (DOPC), GM_1 ganglioside (bovine brain sodium salt), N-stearoyl-D-erythro-sphingosylphosphorylcholine (SM) and cholesterol (ovine wool) were purchased from Avanti Polar Lipids (Alabaster, AL, USA). Sucrose, Bovine serum albumin (BSA) and Phophate Buffered saline (PBS) were purchased from Sigma Aldrich (St.Louis, USA). Organic solvents of spectroscopic grade were purchased from Merck (Darmstadt, Germany). Fluorescent probe Atto565 was coupled to GM_1 as described below.

Preparation of GUVs

Giant unilamellar vesicles (GUVs) were prepared using electroformation in custom-made Teflon chambers equipped with two platinum electrodes [1]. Lipids were dissolved in either chloroform or a 2:1 chloroform/methanol mixture, with fluorescently labeled ganglioside GM1 (GM1-Atto565) added at a probe-to-lipid ratio of 1:5000. A 6 µl volume of this lipid mixture was spread on each electrode and allowed to evaporate. Then, 370 µl of a sucrose solution (300 mOsm/kg) was added to the chamber. The electroformation protocol consisted of applying an alternating electric field (2 V peak-to-peak, 10 Hz) for 1 hour while incubating the samples at 47°C. Afterward, the frequency was reduced to 2 Hz, and the electric field (2 V peak-to-peak) was applied for an additional 30 minutes. The samples were then allowed to cool gradually. In the final steps, 150 µl of BSA was used to coat an 8-well µ-Slide (Ibidi, Munich, Germany). Following this, 50 µl of GUVs and 100 µl of PBS buffer were added to the slide. No special procedures were performed to immobilize the GUVs.

Tagging Atto565 to GM_1

GM_1-Atto565 was obtained by re-acylation of de-acetylated GM_1, received as described in [2]. In brief, 4 mg (2.6 nmol) de-acetylated GM_1 was dissolved in 1 ml of freshly distilled DMF, 1 ml of triethylamine was added and 2 mg (2.9 nmol) of Atto565-N-hydroxysuccinimidyl ester (Sigma-Aldrich), dissolved in 200 µl of MeCN was added. The reaction mixture was stirred on a magnetic stirrer for 96 h at room temperature. The reaction mixture was evaporated and GM_1-Atto565 was isolated by column chromatography, using Silica gel 100 (Merck) in chloroform-methanol-water, 65:25:4 (v/v/v) with a yield of 1.1 mg (0.52 nmol) (14%, M_r = 1997). R_f = 0.45 (TLC chloroform : methanol : H_2O, 60:40:9, v/v/v),

where R_f is substance's mobility in the TLC chromatography, calculated as the fraction of the front mobility.

Simulating two-dimensional probe movement in the membranes containing mobile nanodomains

To generate STED-FCS diffusion law plots for lipid membranes with mobile nanodomains we employed Monte Carlo (MC) simulations. The simulation script is available at **[https://doi.org/10.48700/datst.sg1fq-8rc76].** These simulations modelled the two-dimensional movement of lipid molecules within the membranes, generating fluorescence intensity traces for different focal spot sizes, ranging from 10 to 250 nm. The details of this computational approach are described in [3,4]. Briefly, each Monte-Carlo simulation begins with a random distribution of nanodomains within a square simulation box, where the side length, *b*, ranges from 4000 to 8000 nm depending on the nanodomain size. Diffusion is initiated by updating the positions of the nanodomains in both directions according to $x_i = x_i^0 + \mathrm{randn}\sqrt{2Ddt}$ where x_i and x_i^0 are the new and initial position in *x*, randn is a randomly generated number with normal distribution and *D* and *dt* denote the diffusion coefficient and a time step of the simulation, respectively. In the simulations, the diffusion coefficient of nanodomains was calculated by the Saffman-Delbrück equation, predicting only a weak logarithmic dependence of *D* on the nanodomain radius *R*, confirmed throughout this work, $D = \left[\ln\left(\frac{2L}{R}\right) - 0.577\right] / \left[\ln\left(\frac{2L}{R_{\mathrm{probe}}}\right) - 0.577\right]$. Here, L is a so-called Saffman-Delbrück length, with $L \approx 1000$ nm and R_{probe} a probe radius with $R_{\mathrm{probe}} \approx 0.5$ nm.[4,5] Following this, fluorescent probes are distributed between the nanodomains and the surrounding bilayer based on the distribution constant constant K_d defined as $K_d = \frac{c_p^{\mathrm{in}}}{c_p^{\mathrm{out}}} = \frac{N_p^{\mathrm{in}} A_{\mathrm{out}}}{N_p^{\mathrm{out}} A_{\mathrm{in}}}$ where c_p^{in} and c_p^{out} denote the concentrations of probes inside and outside of the nanodomains and A_{in} and A_{out} signify the total area occupied by the nanodomains or the rest of the bilayer. If a domain collides with another domain or a neighbouring probe, it is reset to its original position. Probes inside and outside the domains diffuse independently. When a probe encounters a domain boundary, a uniformly distributed random number between 0 and 1 is generated and compared to the probability of crossing the boundary, defined by P_{out} (for exiting) and P_{in} (for entering a domain), respectively. These probabilities relate to K_d via $K_d = \frac{P_{\mathrm{in}} f_{\mathrm{out}}^b A_{\mathrm{out}}}{P_{\mathrm{out}} f_{\mathrm{in}}^b A_{\mathrm{in}}}$ where $f_{\mathrm{in/out}}^b$ defines the fraction of the boundary region as the ratio between the boundary area and the total area of the respective region (either the nanodomain interior or exterior). If the generated

random number exceeds P_{in} or P_{out}, the probe crosses the boundary and transitions into the new environment. To enhance statistical accuracy, multiple foci were implemented at different positions within the simulation box. Specifically, simulations with mobile nanodomains used four focal points, whereas those with static nanodomains required ten. The simulations assume that the focused laser beam illuminating the detection volume, as well as the molecular detection efficiency, follow a Gaussian intensity distribution. Consequently, the expected number of photons emitted by each probe during a simulation step is given by $I_i = \text{poissrnd}\left[I_0 \exp\left(-2(x_i^2 + y_i^2)\right)/w^2\right]$. Here, poissrnd[] denotes the Poissonian distribution of photons, and I_0 is the emission density in the centre of the laser focus. Domains and probes continue to move until the final simulation time is reached. The simulation output consists of a detected fluorescence intensity trace.

This fluorescence intensity trace is subsequently auto-correlated in the same manner as the experimentally acquired data to derive STED-FCS autocorrelation functions for different focal spot sizes ($G(\tau, w)$):

$$G(\tau, w) = \frac{\langle \Delta I(t, w) \Delta I(t + \tau, w) \rangle}{\langle \Delta I(t, w) \rangle^2}. \quad \text{(S1)}$$

Here I(t) represents intensity at time t and τ is the lag-time. To determine the probe diffusion coefficient $D(w)$ for each spot size, the autocorrelation curves are fitted using a model that considers anomalous two-dimensional diffusion:

$$G(\tau) = 1 + \frac{1}{N} \cdot \frac{1}{(1 + \frac{\tau}{\tau_D})^{\alpha}} \quad \text{(S2)}$$

Here, τ_D is the diffusion time, representing the average time for a molecule to move from the center to the edge of the focal spot, and it is related to D via $D = \frac{w^2}{4\tau_D}$. In equation 2, N denotes the number of particles within the analyzed volume, and α is the anomalous factor. For free diffusion, $\alpha = 1$, but as diffusion deviates from this ideal, α deviates accordingly [6,7]. Finally, a set of STED-FCS diffusion law plots is constructed by plotting D versus w, combining the parameters outlined in **Table S1** to depict the dependence of the diffusion law plot shape on these variables.

Table S1: Input parameters used for Monte Carlo simulations of probe diffusion in the presence of moving nanodomains. The parameters involve probe diffusion coefficient outside D_{out} and inside D_{in} nanodomains, nanodomain radius R_d,

membrane surface fraction occupied by nanodomains and the probe distribution coefficient between nanodomains and the surroundings K_d. The scheme illustrates the process of parameter variation, with a separate simulation conducted for each listed combination in the table. Parameters highlighted by red squares remained unchanged throughout the scheme unless their specific impact was under investigation. The values for nanodomain diffusion D_d were calculated according to the Saffman- Delbrück model [5].

Par	Value	Diagram
D_{out} (μm²/s)	9	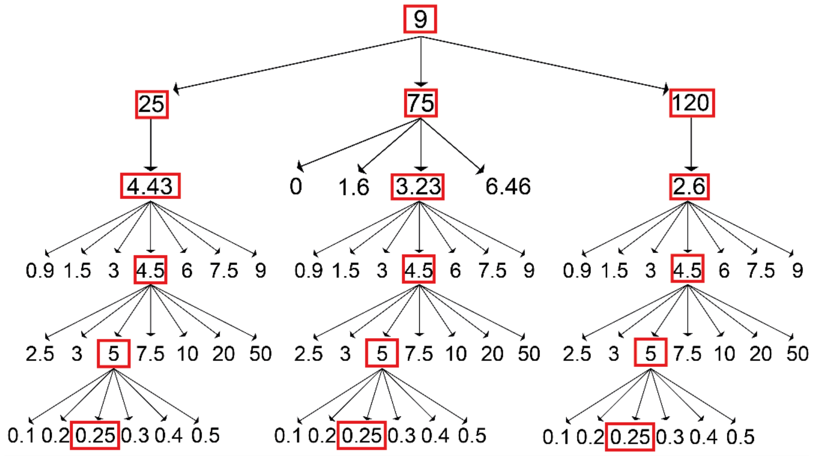
R_d (nm)	25 – 120	
D_d (μm²/s)	2.6 – 4.4	
D_{in} (μm²/s)	0.9 – 9	
K_d (-)	2.5 – 50	
f (-)	10 – 50	

The library of in-silico generated STED-FCS diffusion law plots

The present library of diffusion law plots was developed to provide a comprehensive, centralized collection of in silico-generated STED-FCS diffusion plots under various conditions. Its primary purpose is to support more accurate, quantitative interpretation of experimentally measured diffusion plots, which have so far been analyzed mostly qualitatively. Rather than representing a complete set of all possible STED-FCS diffusion plots, this library serves as a flexible and expandable resource that can be continuously updated with newly generated plots.

The library is implemented as an easy-to-use macro that enables users to quantitatively compare an experimental diffusion law plot—inserted into the macro—with the simulated diffusion dependencies. This comparison is based on the calculation of the reduced chi-squared (χ^2) value, providing a quantitative measure of similarity between the experimental and simulated data. As a result, this approach allows for robust quantitative characterization of experimentally measured diffusion law plots.

A core data set A of diffusion law plots was generated for three fundamental nanodomain sizes: R = 25, 75, and 120 nm. In this data set, other simulation parameters were systematically varied following the scheme outlined in **Table S1**. The initial values used in the

simulations were as follows: D_d, representing diffusion coefficient calculated with the Saffman-Delbrück model; D_{in} = 4.5 μm^2/s, corresponding to half the diffusion coefficient in a DOPC/Chol (25/75) membrane, reflecting a more viscous and ordered environment of the interior of ganglioside nanodomains[8–10]; K_d = 5, indicating moderate probe affinity to nanodomains; and f = 0.25, chosen as a representative mid-range value. If not stated otherwise, D_{out} = 9 ± 1 μm^2/s, corresponding to free probe diffusion in a homogeneous DOPC/Chol membrane was considered. In cases where D_{out} takes other values, all diffusion coefficients can be normalized so that the values of all diffusion coefficients are relatively conserved.

The resulting diffusion plots are shown in **Figure S1**. This data set (located at the top of the library) is supplemented by a data set B (at the bottom), putting together additional diffusion law data, which explore the parameter space more broadly using coarser step sizes, specifically $R \in \{25; 120\}\text{nm}, f \in \{0.1; 0.3; 0.5\}, K_\text{d} \in \{2.5; 5; 10; 20\}, D_\text{in} \in \left\{\frac{D_\text{out}}{3}; \frac{D_\text{out}}{2}; \frac{D_\text{out}}{1.5}\right\}$ and $D_\text{d} \in \{1.3\mu\text{m}^2/s; SD; 5.2\mu\text{m}^2/s\}$ for large nanodomains and $D_\text{d} \in \{SD; 9.5\mu\text{m}^2/s\}$ for small nanodomains. Together, these two core data sets span a wide range of combinations of the parameters R, f, K_d, D_{in} and D_d. This extended coverage enhances the library's applicability to a variety of experimental conditions and enables a coarse estimation of selected parameters — specifically those not fixed in advance—based on the simulated plot that yields the lowest chi-squared value when compared with experimental data. The precision of this estimation is determined by the step sizes used for each parameter (see above as well as **Table S1**). Accuracy can be further improved by extending the library with new plots generated via Monte Carlo simulations of probe diffusion (**[https://doi.org/10.48700/datst.sg1fq-8rc76]**).

Supporting results

Ratiometric parameter		160/60			R_{confin}			S		
Focal spot size (nm)		60-160			60			60-160		
Domain size		Large	Intermediate	Small	Large	Intermediate	Small	Large	Intermediate	Small
Changing D_{in}	0.9	2.02	1.73	1.15	5.46	2.82	1.71	16.8	23.2	7.73
	1.5	1.95	1.76	1.08	5.17	2.74	1.59	16.5	15.1	4.63
	3	1.74	1.49	1.12	4.51	2.14	1.42	14.7	20.8	7.69
	4.5	1.66	1.45	1.09	3.78	1.81	1.27	15.8	22.3	6.53
	6	1.65	1.43	1.08	3.63	2.01	1.32	16.1	19.0	5.76
	7.5	1.33	1.29	1.08	2.75	1.72	1.26	10.9	15.3	5.73
	9	1.21	1.27	1.03	2.34	1.57	1.18	8.02	15.5	2.46
Changing K_d	2.5	1.19	1.20	1.07	1.62	1.26	1.12	10.4	14.3	5.51
	3	1.22	1.32	1.06	2.14	1.58	1.22	9.07	18.1	4.32
	5	1.55	1.40	1.09	3.24	2.02	1.58	15.3	17.7	6.13
	7.5	2.00	1.65	1.02	4.50	2.26	2.02	20.0	25.9	1.20
	10	2.35	1.71	1.10	5.43	2.58	2.58	22.4	24.6	5.62
	20	2.56	1.75	1.04	6.72	2.95	2.95	20.9	22.9	2.42
	50	2.70	1.79	1.01	7.70	3.15	3.15	19.9	22.7	0.58
Changing *f*	10%	1.29	1.41	1.15	3.91	2.48	1.35	5.94	14.8	10.1
	20%	1.46	1.41	1.14	3.57	2.15	1.34	11.5	17.2	9.37
	30%	1.64	1.48	1.03	3.29	1.85	1.37	17.6	23.4	2.21
	40%	1.79	1.38	0.96	2.66	1.69	1.48	26.9	20.3	-2.13
	50%	1.67	1.26	0.94	2.18	1.62	1.70	27.6	14.2	-3.23

Table S2 presents ratiometric parameters $D_{\mathrm{confocal}}/D_{\mathrm{STED}}$ represented by the red color map, R_{confin} by the violet colour map, and *S* by the yellow colour map used as indicators of nanodomain size and fraction. The parameters were calculated for large (R_d = 120 nm), intermediate (R_d = 75 nm), and small nanodomains (R_d = 25 nm), based on the probe diffusion coefficient within the nanodomains (D_{in}), probe partition coefficient between nanodomains and surroundings (K_d), and fraction of nanodomains in the membrane (*f*). If not stated otherwise, D_{in} = 4.5µm^2/s, K_d =5, *f* = 0.25. The colour code illustrates a gradual change from low (light) to high (dark) values.

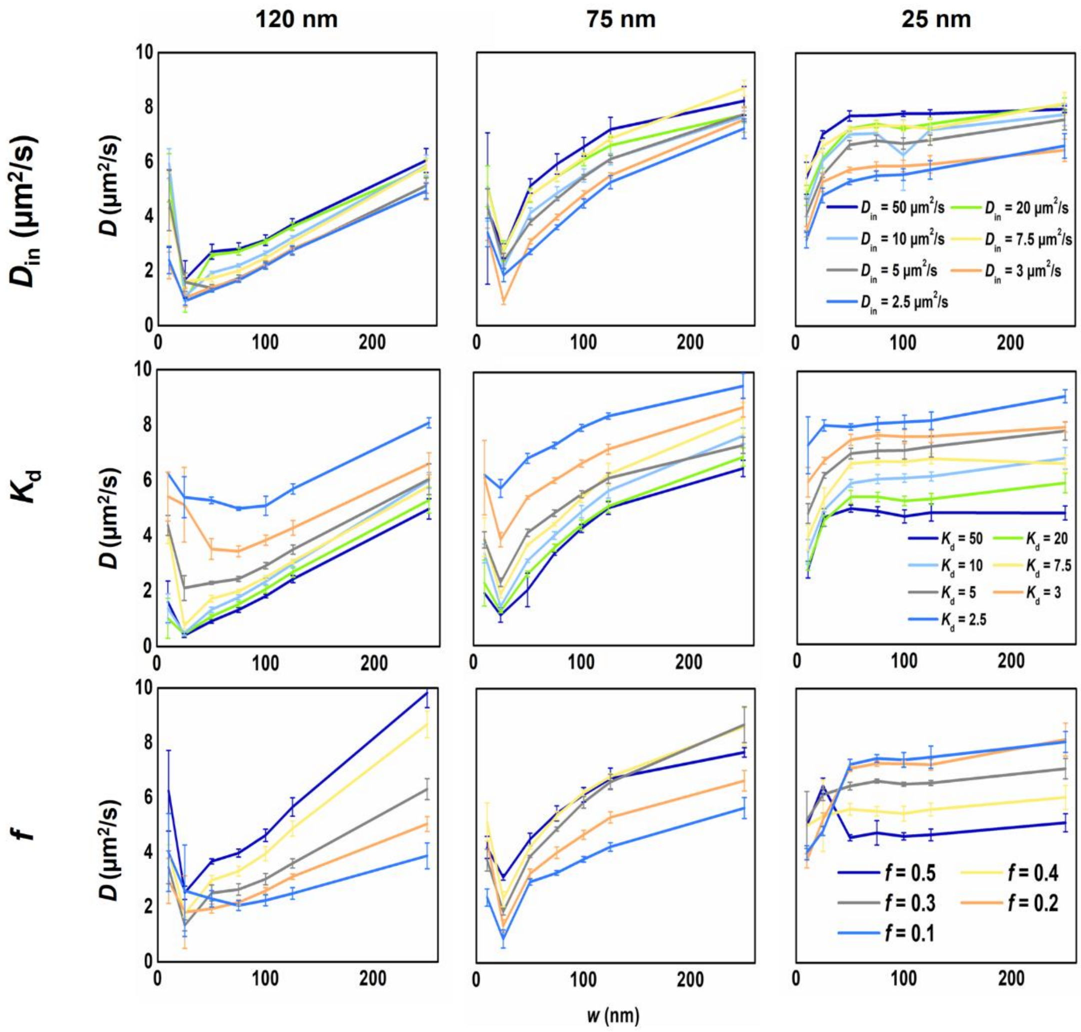

Figure S1: Computationally generated STED-FCS diffusion law plots illustrating mobile nanodomains with D_d modelled according to the Saffman-Delbrück model. Plots are shown for three nanodomain sizes: large (left column), intermediate (middle column) and small (right column). The impact of the probe diffusion coefficient within the nanodomains (D_{in}) is depicted in the upper row, the probe distribution coefficient between the nanodomains and the surroundings (K_d) in the middle row, and the area fraction (f) occupied by nanodomains in the lower row. If not stated otherwise, D_{in} = 4.5μm^2/s, K_d =5, f = 0.25.

Trends in the anomalous coefficient alpha

Building upon the analysis presented in **Figure S1**, we extended our investigation to establish a relationship between the anomaly factor (referred to as alpha) and the size of the confocal spot *w*, termed alpha plot dependency (**Figure S2**). This factor, determined by fitting individual STED-FCS autocorrelation functions by a model accounting for anomalous diffusion (**Eq 2**), equals 1 for free diffusion, while it achieves values less than 1 for anomalous diffusion. Importantly, the resulting dependencies closely resemble the diffusion law plots outlined in **Figure S1**, exhibiting a distinct asymmetric funnel shape. This characteristic shape

thus emerges as an additional indicator of nanodomain size. A potential drawback of this approach might be that achieving the minimum alpha necessitates extremely small confocal spot sizes, which may pose challenges in STED microscopy.

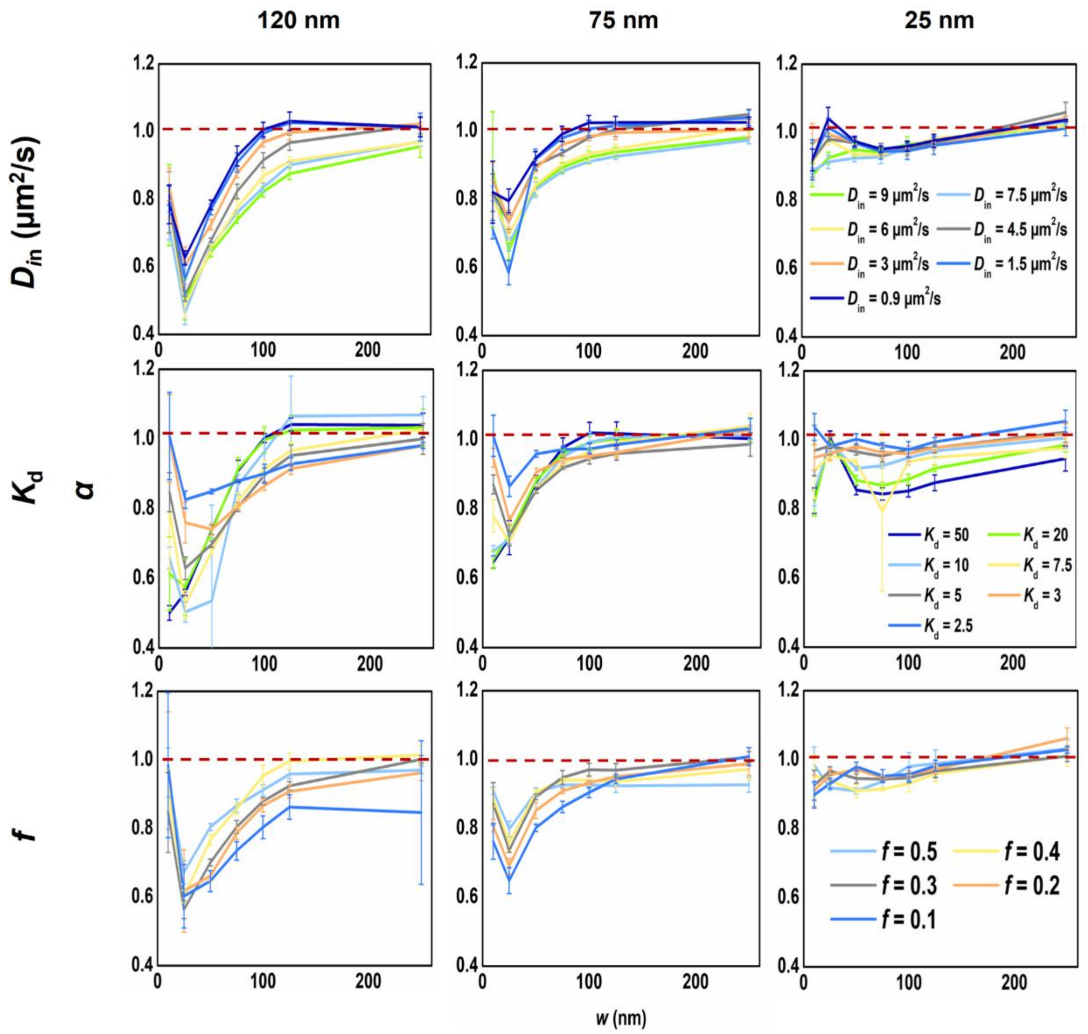

Figure S2: A set of alpha plots complementary to **Figure S1** establishing a relationship between the anomaly factor α and the size of the confocal spot *w*. Alpha plot dependency exhibits a pronounced funnel shape, whereby the depth of this funnel's minimum increases with larger moving nanodomains. For nanodomains with R_d = 120 nm, the minimum alpha falls within the range of 0.4-0.6, while for nanodomains with R_d = 75 nm, the minimum alpha rises to 0.6-0.8. Nanodomains with R_d = 25 nm show a slight decrease in alpha, ranging from 0.9 to 1. The impact of D_{in} is depicted in the upper row *K*d in the middle row, and f lower row. If not stated otherwise, D_{in} = 4.5 μm²/s, K_d =5, f = 0.25.

References

(1) Angelova, M. I.; Dimitrov, D. S. Liposome Electroformation. *Faraday Discuss. Chem. Soc.* **1986**, *81*, 303–311. https://doi.org/10.1039/dc9868100303.

(2) Sonnino, S.; Kirschner, G.; Ghidoni, R.; Acquotti, D.; Tettamanti, G. Preparation of GM1 Ganglioside Molecular Species Having Hemogeneous Fatty Acid and Long Chain Base Moieties. *J. Lipid Res.* **1985**, *26* (2), 248–257. https://doi.org/10.1016/s0022-2275(20)34395-9.

(3) Wawrezinieck, L.; Rigneault, H.; Marguet, D.; Lenne, P. F. Fluorescence Correlation Spectroscopy Diffusion Laws to Probe the Submicron Cell Membrane Organization. *Biophys. J.* **2005**, *89* (6), 4029–4042. https://doi.org/10.1529/biophysj.105.067959.

(4) Šachl, R.; Bergstrand, J.; Widengren, J.; Hof, M. Fluorescence Correlation Spectroscopy Diffusion Laws in the Presence of Moving Nanodomains (2016 J. Phys. D: Appl. Phys . 49 114002). *J. Phys. D. Appl. Phys.* **2016**, *49* (18), 189601. https://doi.org/10.1088/0022-3727/49/18/189601.

(5) Saffman, P. G.; Delbrück, M. Brownian Motion in Biological Membranes. *Proc. Natl. Acad. Sci.* **1975**, *72* (8), 3111–3113. https://doi.org/10.1073/pnas.72.8.3111.

(6) Sezgin, E.; Levental, I.; Grzybek, M.; Schwarzmann, G.; Mueller, V.; Honigmann, A.; Belov, V. N.; Eggeling, C.; Coskun, Ü.; Simons, K.; Schwille, P. Partitioning, Diffusion, and Ligand Binding of Raft Lipid Analogs in Model and Cellular Plasma Membranes. *Biochim. Biophys. Acta - Biomembr.* **2012**, *1818* (7), 1777–1784. https://doi.org/10.1016/j.bbamem.2012.03.007.

(7) Sezgin, E.; Schneider, F.; Galiani, S.; Urbančič, I.; Waithe, D.; Lagerholm, B. C.; Eggeling, C. *Measuring Nanoscale Diffusion Dynamics in Cellular Membranes with Super-Resolution STED–FCS*; 2019; Vol. 14. https://doi.org/10.1038/s41596-019-0127-9.

(8) Koukalová, A.; Amaro, M.; Aydogan, G.; Gröbner, G.; Williamson, P. T. F.; Mikhalyov, I.; Hof, M.; Šachl, R. Lipid Driven Nanodomains in Giant Lipid Vesicles Are Fluid and Disordered. *Sci. Rep.* **2017**, *7* (1), 5460. https://doi.org/10.1038/s41598-017-05539-y.

(9) Sarmento, M. J.; Owen, M. C.; Ricardo, J. C.; Chmelová, B.; Davidović, D.; Mikhalyov, I.; Gretskaya, N.; Hof, M.; Amaro, M.; Vácha, R.; Šachl, R. The Impact of the Glycan Headgroup on the Nanoscopic Segregation of Gangliosides. *Biophys. J.* **2021**, *120* (24), 5530–5543. https://doi.org/10.1016/j.bpj.2021.11.017.

(10) Davidovic, D.; Kukulka, M.; Sarmento, M. J.; Mikhalyov, I.; Gretskaya, N.; Chmelov, B.; Ricardo, J. C.; Hof, M.; Cwiklik, L.; Radek, Š. Which Moiety Drives Gangliosides to Form Nanodomains ? *J. Phys. Chem. Lett.* **2023**, *14*, 5791–5797. https://doi.org/10.1021/acs.jpclett.3c00761.
